# Identification of Chromosomal Genes in *Yersinia pestis* that Influence Type III Secretion and Delivery of Yops into Target Cells

**DOI:** 10.1371/journal.pone.0034039

**Published:** 2012-03-30

**Authors:** Andrew S. Houppert, Elizabeth Kwiatkowski, Elizabeth M. Glass, Kristin L. DeBord, Peter M. Merritt, Olaf Schneewind, Melanie M. Marketon

**Affiliations:** 1 Department of Biology, Indiana University, Bloomington, Indiana, United States of America; 2 Department of Microbiology, University of Chicago, Chicago, Illinois, United States of America; 3 Mathematics and Computer Science Division, Argonne National Laboratory, Argonne, Illinois, United States of America; University of Louisville, United States of America

## Abstract

Pathogenic *Yersinia* species possess a type III secretion system, which is required for the delivery of effector Yop proteins into target cells during infection. Genes encoding the type III secretion machinery, its substrates, and several regulatory proteins all reside on a 70-Kb virulence plasmid. Genes encoded in the chromosome of yersiniae are thought to play important roles in bacterial perception of host environments and in the coordinated activation of the type III secretion pathway. Here, we investigate the contribution of chromosomal genes to the complex regulatory process controlling type III secretion in *Yersinia pestis*. Using transposon mutagenesis, we identified five chromosomal genes required for expression or secretion of Yops in laboratory media. Four out of the five chromosomal mutants were defective to various extents at injecting Yops into tissue culture cells. Interestingly, we found one mutant that was not able to secrete *in vitro* but was fully competent for injecting Yops into host cells, suggesting independent mechanisms for activation of the secretion apparatus. When tested in a mouse model of plague disease, three mutants were avirulent, whereas two strains were severely attenuated. Together these results demonstrate the importance of *Y. pestis* chromosomal genes in the proper function of type III secretion and in the pathogenesis of plague.

## Introduction

*Yersinia pestis*, the causative agent of plague, is one of three *Yersinia* species pathogenic to humans. All three species share a tropism for lymphoid tissues and utilize a type III secretion system (TTSS) to undermine the host immune system [1], [2]. The *Yersinia* TTSS is encoded by a conserved 70-kb virulence plasmid, termed pCD1 in *Y. pestis* [3]#. In addition to the machinery components and its secreted substrates (YopB, YopD, YopE, YopH, YopM, YopN, YopO, YopP, YopK, YopR, YopT, LcrV, and LcrQ), pCD1 encodes several regulatory proteins that respond to environmental cues. Expression of the TTSS and its substrates are induced upon temperature shift to 37°C by the transcriptional activator LcrF [4], [5], [6], [7]. YopD, LcrH, and YscM1/YscM2 (LcrQ) repress activation of the TTSS in the absence of certain serum amino acids [8]#. YopN, TyeA, SycN, YscB and LcrG prevent transport of Yop effectors until contact with a target cell is made [9], [10], [11], [12], [13], [14], [15], [16], [17], [18]. These conditions can be simulated *in vitro*, and upon a shift to 37°C in the absence of calcium, yersiniae undergo growth cessation and massively secrete all of the type III substrates into the medium [19], [20], [21], [22], [23]. This phenomenon is referred to as the LCR (low calcium response) phenotype [24]#. The onset of bacteriostasis under TTSS-inducing conditions is particularly severe for *Y. pe*stis compared to the enteric *Yersinia* and correlates with a loss of aspartase and glucose-6-phosphate dehydrogenase functions [19], [25], [26], [27]. Interestingly, calcium does not prevent growth or Yop expression during cell culture, and indeed bacteriostasis can be uncoupled from induction of the *Yersinia* TTSS by manipulating levels of glutamate, sodium, and pH to reflect the presumed *in vivo* conditions of a necrotic lesion [19], [28], [29], [30]. Therefore while the calcium-dependent LCR phenotype may be an artifact of *in vitro* culture conditions, this *Yersinia*-specific phenomenon has been utilized as a tool to investigate the details of TTSS regulation and function in *Yersinia*. It has been suggested that yersiniae sense environmental calcium concentrations in the extracellular fluids of their host and in the cytoplasm of host cells via insertion of the type III secretion needle into the plasma membrane of target host cells [16], [31], [32]; however the mechanism for sensing calcium levels remains a mystery.

Although many of the genes required for activation of the type III pathway in response to environmental signals have been identified, the mechanism by which yersiniae perceive their environment and contact with a target cell and then transmit that information to the cytoplasm and secretion apparatus remains unclear. Also, early work in *Y. enterocolitica* suggested that the virulence plasmid is necessary, but not sufficient, for expression and/or delivery of effector Yops [33], [34]#. Presumably, additional unidentified genes are involved in the regulation of type III secretion. In 1984, Goguen and colleagues performed a genetic analysis, taking advantage of the LCR growth phenotype that occurs at 37°C in the absence of calcium, and reported that a large number of *Y. pestis* mutations, which seemed to be chromosomally located, gave rise to an LCR^−^ phenotype [24]. However, these mutants remain uncharacterized.

In an effort to determine the potential role of chromosomal genes in the regulation of the *Yersinia* TTSS, we undertook a screen similar to that of Goguen *et al*. Using a Mu transposon derivative [35], we generated insertional mutations in *Y. pestis* and isolated mutants with an LCR^−^ phenotype, i.e. the variants gained the ability to grow at elevated temperatures in the absence of calcium. These mutants were then evaluated for their ability to secrete Yops when grown in calcium-depleted medium. The location of the transposon insertion was determined for each secretion-defective mutant based on inverse PCR and sequence analysis. After screening 2067 mutants displaying the LCR^−^ growth phenotype, we identified 5 variants (termed CHI strains for chromosomal insertion) harboring a transposon insertion that gave rise to defects in secretion of Yops and/or expression of the TTSS. The ability of the 5 strains to target Yops into host cells during tissue culture infection was also analyzed, revealing a distinction between a secretion defect and a targeting defect in some cases. Further characterization of the mutants in a mouse model of infection confirmed the importance of the identified genes in virulence.

## Materials and Methods

### Bacterial strains and media

All *Yersinia pestis* strains are attenuated (non-pigmented) variants of *Y. pestis* biovar *mediaevalis* KIM lacking the 102-kb *pgm* locus [36]. KIM5 (pCD1+, pMT1+, pPCP1+) was obtained from R. Brubaker. KIM8 (pCD1+, pMT1+, pPCP1−) and YP769 (also called KIM8 Δ1234 [37]; pMT1+, pPCP1−, pCD1+ Δ*yopM/yopT/sycT/yopK/ylpA/yopE/sycE/ypkA/yopJ/yopH*) were obtained from G. Plano. *Y. pestis* strains were propagated on Heart Infusion or Blood Base agar plates at 26°C for two days. Overnight cultures were grown in Heart Infusion Broth (HIB) at 26°C. Antibiotics were added as appropriate to a final concentration of 20 µg/ml chloramphenicol, 50 µg/ml ampicillin, or 50 µg/ml kanamycin. *Escherichia coli* strains were propagated in LB broth or agar, supplemented with 20 µg/ml chloramphenicol, 100 µg/ml ampicillin, or 25 µg/ml kanamycin, at 37°C, except CR202, which was grown at 30°C.

### Mu Mutagenesis

*E. coli* CR202 carries the Mu*cts*62pAp5hP1#1 lysogen (also called MuAphP1), a Mu-P1 hybrid [35]#. To obtain a lysate, overnight cultures of CR202 were subcultured 1∶50 into HIB, supplemented with 2.5 mM MgCl_2_ and 2.5 mM CaCl_2_, and incubated at 30°C for 1 hour 45 minutes. The culture was then shifted to 42°C for 2 hours, or until lysis occurred. The lysed culture was sterilized by passing through a 0.2 µm filter and then stored on ice until needed. *Y. pestis* KIM5 overnight cultures were diluted 1∶25 into the same medium and incubated at 26°C until late exponential phase, about 3 hours. Cells were sedimented by centrifugation and suspended in 10 mM MgSO_4_, followed by incubation at room temperature for 20 minutes. To infect, 5 ml of lysate was added to 2.5 ml of cells and incubated at room temperature for 20 minutes. The cells were again sedimented by centrifugation, suspended in 10 mM MgSO_4_, and plated on Blood Base agar containing 20 mM sodium oxalate, 20 mM MgCl_2_ (BB/MgOx agar), and 50 µg/ml ampicillin to select for Mu insertions leading to an LCR^−^ phenotype. During initial testing, the plates were incubated at a range of temperatures (26°C, 30°C, 33°C, 35°C, and 37°C) for 2 days. At temperatures above 33°C, many chromosomal insertion mutants also carried additional Mu insertions on pCD1. We therefore chose 33°C for routine selection of LCR^−^ mutants.

### Identification of mutants

Primer sequences are listed in Table S1. To determine the location of the Mu insertion, chromosomal DNA of mutants was digested with either Taq^α^ I or BspHI, followed by large-volume ligation. Ligations were purified and used as the template for inverse-PCR reactions using primers complimentary to the left (BfaI-LT, BfaI-RT) and right (TaqI-LT, TaqI-RT) ends of Mu. PCR products were sequenced using nested primers. The obtained sequences were subjected to the following computational analysis. Sequences were formatted into FASTA format, using only the transposon sequence plus the following 160 nucleotides. If no transposon sequence was found, the first 260 nucleotides were used. These formatted sequences were then used to search the *Y. pestis* KIM genome using the BLAST algorithm [38]. Positions of significant BLAST hits against the genome were obtained from the BLAST output and the genes surrounding or overlapping these positions were recorded. Significant hits were those with an e-value below 10^−7^ and percent identity over 30. Functional characterization of disrupted genes was carried out using bioinformatic analysis [39], [40], [41].

### Complementation of mutants

False positive mutants were eliminated by pCD1 displacement. *Y. pestis* KIM-3001.P39 carries a tagged pCD1 (referred to here as pCD1::KM) in which the *yopE-sycE* locus was replaced by a kanamycin resistance cassette [42]. Plasmid DNA isolated from KIM-3001.P39 was electroporated into each chromosomal mutant. Transformants were plated on BB containing kanamycin to select for the tagged pCD1 (pCD1::KM). Displacement of the original pCD1 by pCD1::KM was confirmed by immunoblotting to demonstrate the loss of *yopE* and the presence of *npt*. Mutants carrying pCD1::KM were screened by secretion assay and immunoblotting (described below). Mutants retaining the secretion defect were then subjected to gene-specific complementation. Table S1 lists the primers and vectors used to clone and introduce the wild-type gene or operon of interest into each mutant. The PCR products for *y0447 and rfaL* were cloned into the pDONR-221 vector using the BP Clonase mix as per manufacturer's instructions (Invitrogen) and then transformed into DH5α. The *pgsA* locus was amplified with the pgsAORFNdeI pgsAORFBamHI primers to clone the open reading frame as an NdeI-BamHI fragment. The native promoter was amplified using the uvrYproEcoRI and uvrYproNdeI primers. The EcoRI-NdeI promoter fragment and NdeI-BamHI ORF fragment were cloned into the EcoRI-BamHI site of pHSG576 [43] by three-way ligation. The regions containing *folD* and *pssA* were amplified using the folD.SOE.3, folD.SOE.BamHI.4, pssA.SOE.3, and pssA.SOE.revEcoRI primers. The promoter region encompassing ∼300 bp upstream of *nptII* was amplified using the npt.pro.SOE.PstI. and npt.pro.folD.SOE.2 or npt.pro.pssA.SOE.2 primer pair. The *npt* promoter product and *folD* and *pssA* products were stitched together using the folD.SOE.3 and folD.SOE.BamHI.4 or the pssA.SOE.3 and pssA.SOE.revEcoRI primer pair. The resulting PCR products were cloned into the SmaI site of pBluescript SK+ and sequenced. The P*_npt_*-*folD* and P*_npt_*-*pssA* products were then cloned into the PstI and BamHI or PstI and EcoRI sites of pHSG576. The resulting plasmids were named pAH191 and pAH195. All constructs were introduced into their respective CHI strains by electroporation.

### Standard Secretion Assays

For routine screening, overnight cultures of *Y. pestis* strains were diluted 1∶40 into modified M9 (MM9) and secretion assays were performed as previously described [44]. Briefly, cells were grown at 26°C for 2 hours and then at 37°C for 3 hours. The OD_600_ of each culture was taken following the incubation at 37°C. Cells were sedimented and proteins in the pellet and supernatant fractions were TCA precipitated and visualized by immunoblotting. Samples were loaded onto SDS-PAGE gels based on the OD_600_ of each culture compared to wild type unless noted otherwise. Additional characterization of secretion phenotypes were conducted by diluting overnight cultures into either HIB, supplemented with 5 mM CaCl_2_ or 5 mM EGTA, or DMEM tissue culture medium (see below) with or without supplemented 5 mM EGTA.

### Flow cytometry to measure Bla reporter injection

Plasmids expressing YopM-Bla (pMM83), Gst-Bla (pMM91), and YopE-Bla (pMM85) from their native promoters have been previously described [44]. pAH83 was created to drive constitutive expression of YopM-Bla. The regions containing *yopM* and *bla* were amplified from pCD1 and pMM83, respectively, using the npt.pro.yopM.SOE.3 and yopM.SOE.4.bla.2 or yopM.SOE.4.bla.2 and bla.SOE.4.EcoRI primer pair. The *nptII* promoter was amplified using the npt.pro.SOE.BamHI.1 and npt.pro.yopM.SOE.2 primers. The resulting *yopM*, *bla*, and *npt* promoter products were stitched together with Phusion using the primers npt.pro.SOE.BamHI.1 and bla.SOE.4.EcoRI. The resulting PCR product was cloned into the SmaI site of pBluescript SK+. The P*_npt_*-*yopM*-*bla* region was then subcloned into the BamHI and EcoRI sites of pHSG576, creating pAH83. All plasmids were introduced into *Y. pestis* strains by electroporation. The night before the experiment, 3.25×10^5^ CHO (ATCC, CCL-61) cells per well were seeded into a 12 well plate and maintained in F12K supplemented with 10% FBS at 37°C+5% CO_2_. *Y. pestis* strains were grown to mid-exponential phase at 26°C in HIB with chloramphenicol and then pre-induced at 37°C for 1.5 hrs. CHO cells were counted and bacteria were added at an MOI of 10, followed by centrifugation at 500×g for 5 min to facilitate cell contact. The infections were incubated at 37°C for 3 hrs after which the cells were washed with PBS and trypsin was added to remove CHO cells from the plate. The trypsin was quenched with the addition of 1 ml of F12K+10% FBS, cells were pelleted at 500×g for 5 min, and the cell pellets resuspended in 300 µL of HBSS-Flow (1× Hank's Buffered Saline Solution, 0.5 mM EDTA, 25 mM HEPES, 2% BSA, pH 7.4). 50 µL of 2× CCF-2AM+probenicid was added to the cells, and the cells were incubated at room temp for 15 min. Following the incubation, 300 µL of HBSS-Flow was added to each tube, and the cells were analyzed on a BD LSRII flow cytometer to quantitate blue and green fluorescence as previously described [44], [45]. Significance was determined using one-way ANOVA with a Tukey post-test in GraphPad Prism 5.

### Cytotoxicity Assays

The night before the infection, 2.5×10^4^ HeLa cells were seeded into 12 well plates and maintained in DMEM supplemented with GlutaMAX and 10% FBS at 37°C+5% CO_2_. *Y. pestis* strains were grown to mid-exponential phase at 26°C in HIB with ampicillin and then pre-induced at 37°C for one hour. The HeLa cells were washed twice with 1×PBS and counted. The media was replaced with 1 mL of warm DMEM+10% FBS and GlutaMAX containing bacteria at an MOI of 10, followed by centrifugation at 500×g for 5 min to facilitate cell contact. After a 30 minute incubation at 37°C, the medium was removed and replaced with 1 mL of fresh pre-warmed medium and the cells were returned to 37°C for 3.5 hours. Cells were then imaged using an Olympus 1×71 microscope and Olympus MicroSuite Five imaging software. For each well of the triplicate infections, five fields of view were counted (100 cells/field of view) and the average percentage of round cells was determined. The experiment was performed in triplicate and repeated twice. Significance was determined using one-way ANOVA with Tukey post-hoc test in GraphPad.

### Trimethoprim Assays

To evaluate the affect of trimethoprim on type III secretion, secretion assays were performed in MM9. Bacteria were grown at 26°C for 2 hours and then at 37°C for 3 hours. 0, 15, or 30 µg/ml of trimethoprim was added when the cultures were shifted to 37°C. Cells were sedimented and proteins in the pellet and supernatant fractions were TCA precipitated and visualized by immunoblotting. Because YP769 (KIM8 Δ1234) lacks all effector Yops, we introduced the YopM expressing plasmid, pMM207, so that we could track secretion of at least one effector. pMM207 was constructed by amplifying a ∼3.8 kb region containing YopM using the primers YopM-upstream and YopM-downstream, followed by cloning the blunt PCR product into the SmaI site of pUC19. pMM207 was introduced into KIM8 and YP769 by electroporation.

To assess the effect of trimethoprim on Yop injection, HeLa cells (ATCC, CCL-2) were infected with *Y. pestis* strains carrying pMM85 (YopE-Bla). Infections and microscopy were carried out as previously described [44], except that trimethoprim (500 µg/ml) was added during the infection.

### Mouse infections

This study was carried out in accordance with the recommendations in the Guide for the Care and Use of Laboratory Animals of the National Institutes of Health. The work was approved by the Institutional Animal Care and Use Committees at the University of Chicago and Indiana University. All efforts were made to minimize suffering of the mice.*Y. pestis* overnight cultures were diluted 1∶20 into HIB and grown for 3 hours at 26°C. Cultures were washed and diluted to appropriate densities in PBS. 6–8 week old female BALB/c mice (Charles River Laboratories, Inc., Wilmington MA or Harlan Laboratories, Indianapolis IN) were anesthetized with either avertin (∼400 mg/kg) or a cocktail of ketamine (100–130 mg/kg) and xylazine (3–6 mg/kg) intra-peritoneally. Mice were then injected via the retro-orbital plexus with 0.1 ml suspensions of bacteria. The administered doses consisted of serial dilutions from 10^0^ to 10^5^ cfu for KIM5 and 10^2^ to 10^7^ cfu for each mutant. All mice were monitored for signs of disease for 14 days or until acute disease (hunched posture, severely matted fur, emaciation, immobility) was apparent, at which time mice were euthanized by CO_2_ inhalation followed by cervical dislocation.

## Results

### Isolation of LCR^−^ mutants

**Figure 1:**
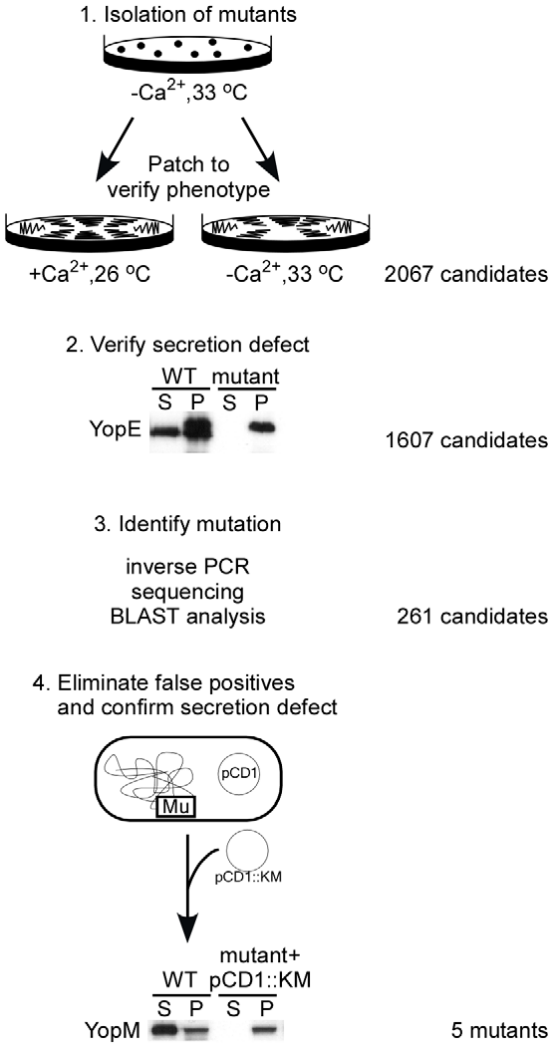
Experimental strategy used to identify *Y. pestis* chromosomal genes involved in type III secretion of Yops. In Step 1, transposon mutants were selected based on their ability to grow at 33°C in the absence of calcium. These mutants were subjected to a secondary screen (Step 2) evaluating their ability to secrete Yops. All mutants that displayed a defect in the synthesis or secretion of Yops were then sequenced to identify the location of the Mu insertion (Step 3). To eliminate mutants that carried unmarked mutations on the virulence plasmid, the tagged pCD1::KM was used to displace the original pCD1, and the transformants were then evaluated for their ability to secrete Yops (Step 4). Strains which retained the secretion defect after Step 4 were kept for further characterization.

The design of the screen to identify chromosomal genes involved in type III secretion of Yop proteins is shown in Figure 1. The parent strain (KIM5) used in this study carries a functional *pla* gene, which encodes a protease known to degrade fibrin clots *in vivo* and is important for infection [46], [47]. We chose to use this strain, rather than a *pla* mutant, so that the transposon mutants could be assayed *in vivo* as well as *in vitro*. Step 1 of the screen was carried out similar to that of Goguen *et al.* [24]#. *Y. pestis* strain KIM5 was mutagenized with Mu*cts*62pAp5hP1#1, a Mu-P1 hybrid also called MuAphP1 [35]. This Mu derivative carries an ampicillin resistance cassette to select for insertions of the transposon into the *Y. pestis* genome. The pool of mutagenized *Y. pestis* cells was then plated directly onto Blood Base agar containing oxalate (a calcium chelator), and ampicillin to select for mutants unable to undergo growth restriction (an LCR^−^ phenotype) at elevated temperatures. We found a distribution of insertions on pCD1 within the *lcrA* (*yopN*, *tyeA*, *sycN*, *yscX*, *yscY*, *lcrD*/*yscV*, *lcrR*), *lcrB* (*yscN*-*U*), *lcrC* (*yscA*-*L*, *lcrQ*), *virF*, and *virG/yscW* loci, demonstrating the validity of the screening process. More importantly, mapping of the insertions by inverse PCR showed that for the majority of chromosomal mutants identified under these conditions there was only one insertion site, indicating that the mutants harbored only a single Mu insertion in the genome.

### Screen for secretion defects

Transposon mutants that were able to grow under inducing conditions (called CHI strains for chromosomal insertion) were subjected to a secondary screen to analyze their ability to secrete Yops *in vitro* (Step 2 in Figure 1). We first evaluated the ability of the KIM5 parent to secrete Yops in various media over different periods of time (data not shown). We chose to use a modified version of M9 (MM9), a minimal medium previously shown to induce secretion of Yops in *Y. enterocolitica* [48] because it gave the highest level of Yop expression and secretion after three hours at 37°C. Characteristic patterns of degraded Yops were observed, due to the Pla protease [49], [50], and these patterns were consistent during repeat experiments. After analyzing 2067 mutants showing a growth phenotype in step 1 of the screen, we identified 1607 strains with decreased secretion, no secretion, or no expression of YopE.

### Locating transposon insertions

Genomic DNA from transposon mutants was used as template for inverse PCR amplification of DNA regions surrounding the Mu insertion. DNA sequences of amplified fragments were subjected to BLAST analysis to locate the position of the insertion (Step 3 in Figure 1). Out of 1607 candidates showing a defect in secretion or expression of YopE, sequences were obtained for 1591 strains. In all but 290 strains, insertions on pCD1 were identified. We were unable to determine the location of insertion for 16 strains. 13 strains harbored insertions in the 100-kb plasmid pMT1 and were not pursued further. The remaining 261 strains carried at least one chromosomal Mu insertion.

**Figure 2:**
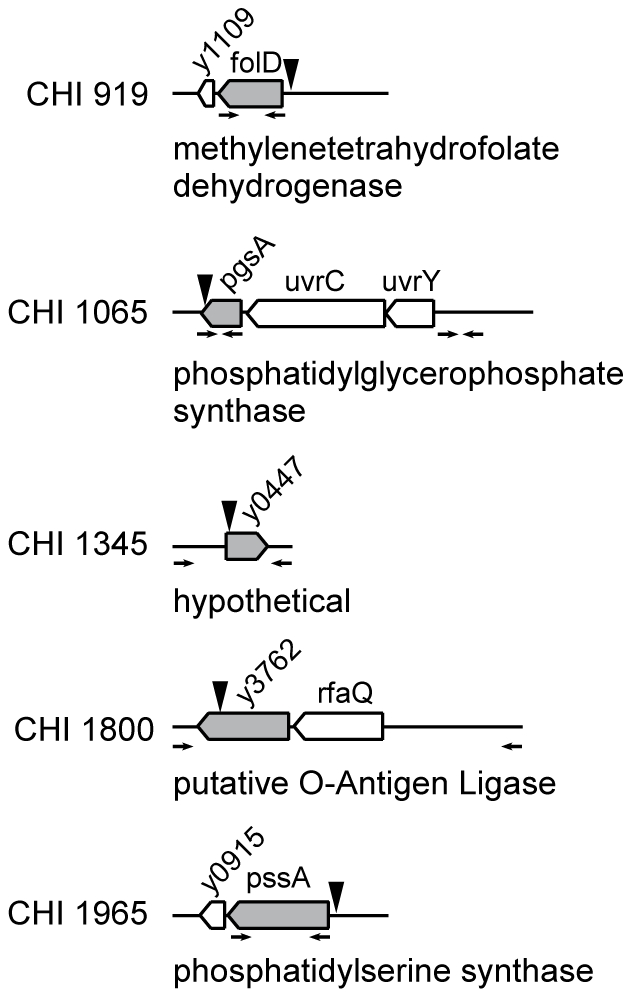
Identification of mutations in CHI strains. The genetic locus carrying the Mu insertion, indicated by an arrowhead, is depicted for each CHI strain. The genome annotations of the disrupted genes (shaded) are shown. Arrows refer to primer locations used to amplify and clone each locus for complementation analysis. Expression of *folD* and *pssA* were driven by the *nptII* promoter.

To verify that the mutant phenotypes were not due to unmarked spontaneous mutations on pCD1, which would not have been identified by our sequencing analysis, we obtained a pCD1 derivative in which the *nptI* cassette conferring kanamycin resistance replaces the *yopE-sycE* locus [42]. This plasmid, which we refer to here as pCD1::KM, was introduced into each of the CHI strains carrying chromosomal insertions (Step 4 in Figure 1). Candidates were screened for their ability to secrete YopM. The replacement of the original pCD1 by the tagged pCD1::KM was verified by immunoblotting to demonstrate the loss of *yopE* and the presence of *nptI* (data not shown). Transposon mutants which regained the ability to secrete YopM after acquiring the new pCD1::KM were discarded. This final step of the screen identified 5 strains that carried a chromosomal insertion leading to a defect in expression and/or secretion of Yops. Arrowheads in Figure 2 indicate the locations of the transposon insertions in these 5 CHI strains. The mutants were next evaluated for their ability to secrete Yops *in vitro*, inject Yops into mammalian cells, and cause disease in a mouse model of infection.

### Secretion phenotypes of CHI strains

**Figure 3:**
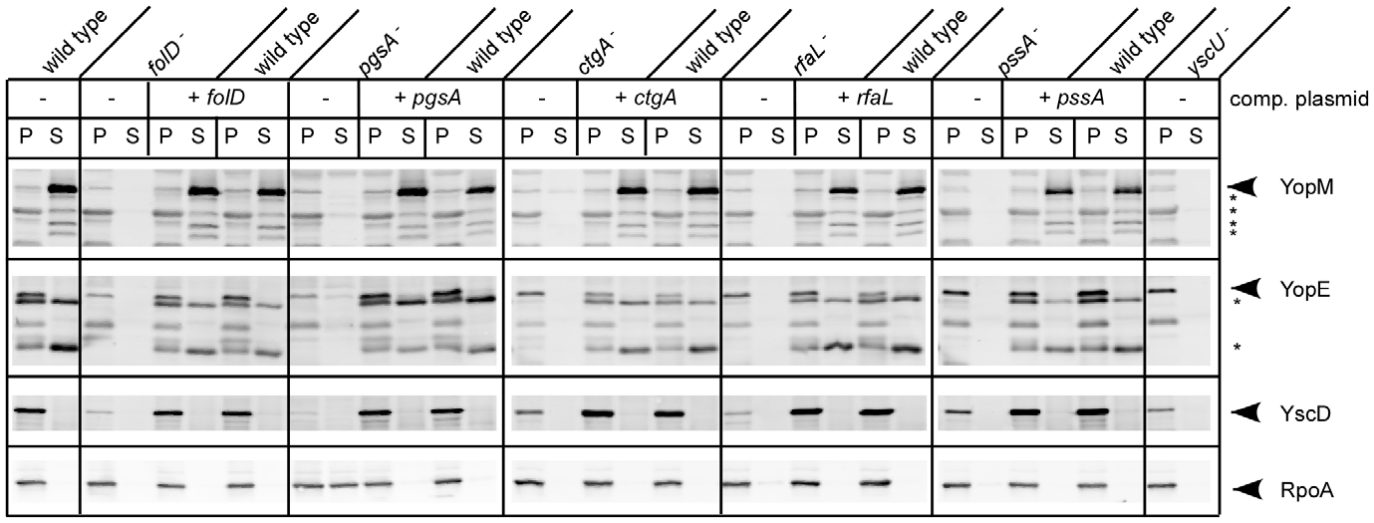
Secretion phenotypes of CHI strains. Complementation plasmids were introduced into each mutant and the KIM5 parent, and the strains were evaluated by performing secretion assays. The TTSS mutant, *yscU*, was included as a negative control. *Y. pestis* strains were grown in MM9 at 26°C for 2 hours and then shifted to 37°C for 3 hours to induce secretion. Proteins in the supernatant (S) and pellet (P) fractions were precipitated and visualized by immunoblotting with antibodies to YopM, YopE (TTSS secreted proteins), YscD (TTSS apparatus component) and RpoA (RNA Polymerase alpha subunit, loading and fractionation control). An asterisk indicates truncated Yops due to degradation by Pla protease.

To characterize each of the transposon mutants, secretion assays were conducted using minimal (MM9) and rich laboratory media (HIB with or without EGTA) (Figure 3 and data not shown). RpoA, which is the alpha-subunit of RNA polymerase, was used a fractionation and loading control, since its expression should be independent of the TTSS and it remains in the bacterial cytoplasm. YscD is a TTSS machine component, which is also not secreted and should only be visible in the cell pellets. To evaluate Yop secretion, we used antibodies against the effectors YopM and YopE. A transposon mutant carrying an insertion in the TTSS component *yscU*, which was isolated from this screen, was included as a negative control. As expected, this mutant did not secrete Yops and displayed lower levels of TTSS proteins. Interestingly, one of the chromosomal mutants displayed a conditional phenotype depending on the medium used in the assay. CHI 1345 (*ctgA^−^*) consistently secreted less Yops than the KIM5 parent strain when grown in MM9 (Figure 3). However, in HIB there was a clear absence of Yops in the culture supernatant for the mutant, in addition to decreased expression of TTSS proteins (not shown). The secretion phenotypes of the remaining CHI strains were consistent in both types of media: they did not secrete YopE or YopM in either MM9 or HIB lacking calcium (Figure 3 and data not shown). Similar to the *yscU* mutant, the chromosomal mutants all had lower expression levels of TTSS related proteins, although to varying degrees. The lack of Yop secretion in these mutants therefore may be due to impaired TTSS expression. When plasmids carrying the respective wild type genes were introduced into each mutant, expression and secretion of Yops was restored, thereby confirming that the mutant phenotypes are the result of the transposon insertions (Figure 3).

### Targeting phenotypes of CHI strains

**Figure 4:**
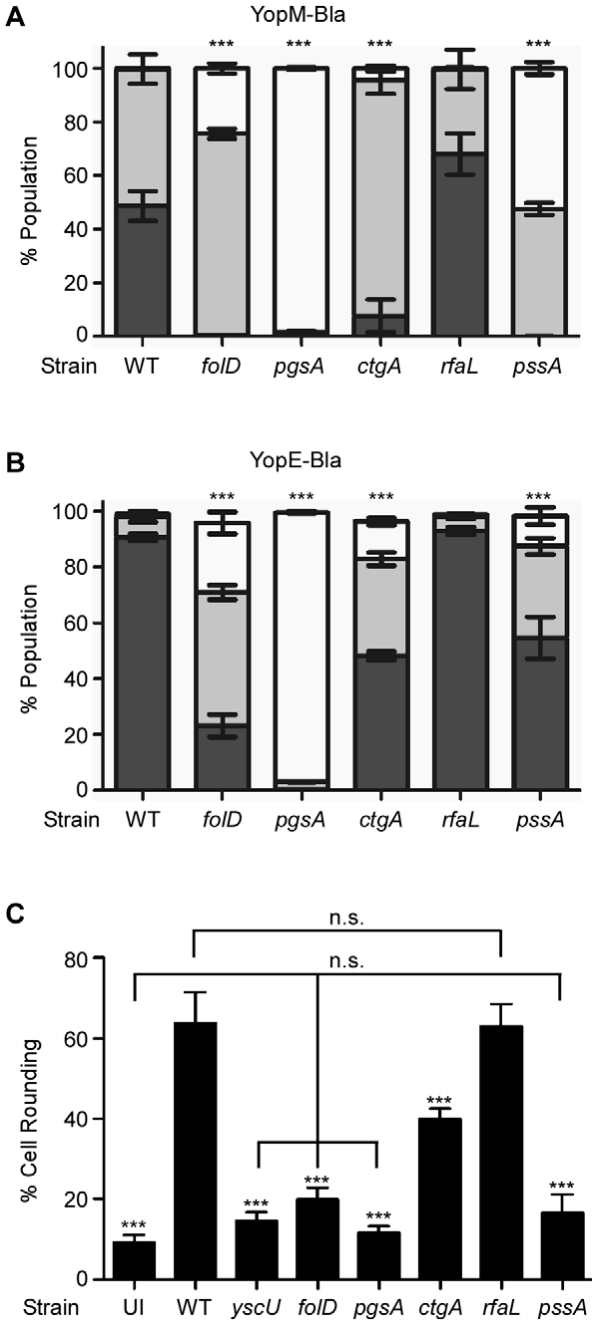
Translocation phenotypes of CHI strains. CHO cells were infected with *Y. pestis* strains carrying either pMM83 (YopM-Bla, Panel A), pMM85 (YopE-Bla, Panel B) or pMM91 (Gst-Bla, not shown) at 37°C for 3 hours, followed by CCF2-AM staining and flow cytometry to quantify blue and green fluorescence. The level of translocated Bla reporter is indicated by white (no injection, green fluorescence), gray (intermediate level of injection, aqua fluorescence), or black (high level of injection, blue fluorescence) bars. For each experiment, the assays were performed in triplicate, and error bars indicate the standard deviation. The data shown is representative of three independent experiments. One-way ANOVA with Tukey post-hoc test was done to demonstrate significant differences in injection (high-level injection, black bars) for mutants relative to the wild type parent. *** *P*<0.001. To assess cytotoxicity due to Yop delivery, cell rounding was also evaluated (Panel C). HeLa cells were infected with the indicated *Y. pestis* strains for 3–4 hours. The percentage of round cells was determined for each infection. The infections were performed in triplicate and error bars represent standard deviation. The data shown is representative of two independent experiments. One-way ANOVA with Tukey post-hoc test was done to determine significance. *** Represents significant difference of mutants compared to WT (*P*<0.001); n.s. = no significance between indicated strains.

The purpose of the *Yersinia* TTSS is to inject effector Yops into target host cells during infection. To determine if any of the transposon insertions identified in our screen contribute to this aspect of virulence, we analyzed the fate of two injected effector Yops (YopE and YopM) during infection of tissue culture cells. We introduced plasmids bearing β-lactamase fusions to YopM (pMM83), YopE (pMM85), or glutathione-S-transferase (Gst, pMM91) [44] into the CHI strains and the KIM5 parent (Figure 4). These strains were used to infect CHO cells at an MOI of 10 for three hours. CCF2-AM, a fluorescent substrate for β-lactamase, was added to stain CHO cells prior to flow cytometry. Cells that had not been injected with β-lactamase hybrids fluoresce green as a result of fluorescence resonance energy transfer (FRET) within intact (uncleaved) CCF2-AM substrate. In the presence of β-lactamase, however, FRET is disrupted, resulting in blue fluorescence of cells that are injected with hybrid Yop-Bla fusions via the *Yersinia* type III pathway. The levels of fluorescence in each cell can be quantified by flow cytometry, and when synchronized, infections by different strains can be quantitatively compared [44], [45]. As previously demonstrated [44], [45], an abundance of blue cells were observed when KIM5 carrying either pMM83 (YopM-Bla) or pMM85 (YopE-Bla), but not pMM91 (Gst-Bla), was used to infect CHO cells (Figure 4 A and B and data not shown). Also as previously demonstrated, the *yscU* ^−^ mutant did not translocate YopM-Bla or YopE-Bla (data not shown). *pgsA^−^* had an identical phenotype to *yscU* ^−^ in that it also did not inject either Yop-Bla reporter into host cells. Surprisingly, *rfaL^−^* injected both Bla reporters at wild type levels. In addition, the remaining mutants all injected YopM-Bla and YopE-Bla, albeit at significantly lower levels than wild type (*** *P*<0.001).

To confirm the translocation phenotypes of the mutants we also evaluated the ability of each mutant to cause cytotoxicity associated with Yop delivery. Toward that end, HeLa cells were infected with *Y. pestis* strains for 3–4 hours and then visualized by microscopy to determine the percentage of cell rounding. As shown in Figure 4C, the majority of cells infected with KIM5 were round, while cells infected with *yscU* ^−^ appeared similar to uninfected cells. Likewise, cells infected with *folD^−^*, *pgsA^−^*, and *pssA* ^−^ all showed no significant difference compared to uninfected cells. The *ctgA* mutant was still able to cause some cytotoxicity, though the percentage of cell rounding was still lower than WT (P<0.001). In contrast, the *rfaL* mutant behaved identically to WT. These data correlate nicely with the Bla reporter assays and confirm that the *rfaL* mutant is able to deliver Yops as well as WT, whereas Yop injection by the remaining mutants is impaired.

### Heterologous expression cannot bypass the mutant defects

**Figure 5:**
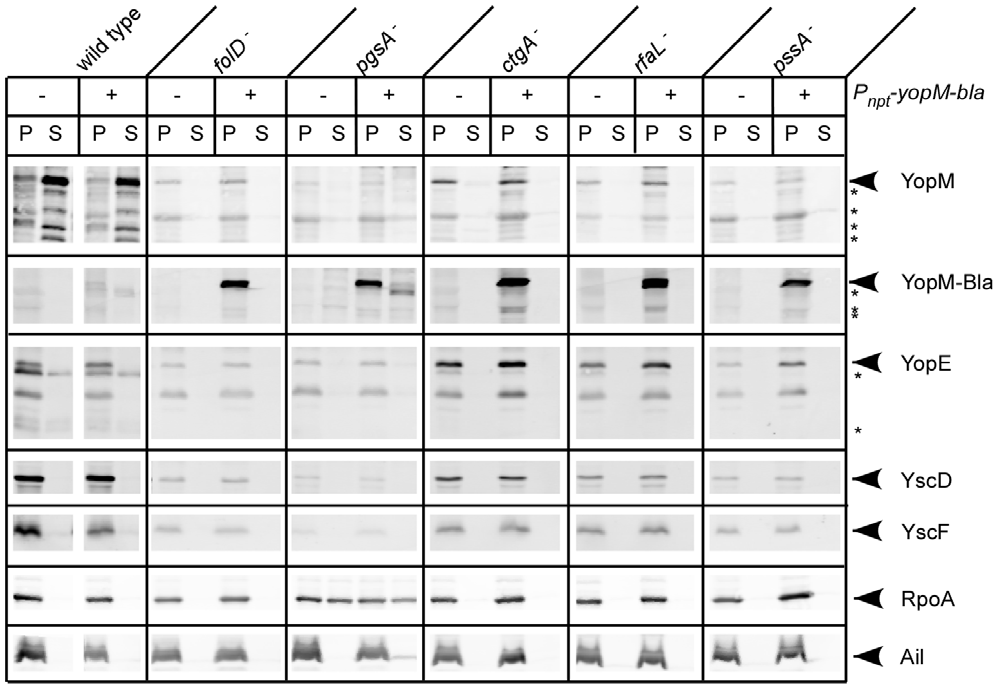
Secretion phenotypes of strains carrying a heterologously expressed Yop reporter. pAH83, which constitutively expresses YopM-Bla, was introduced into each mutant and the KIM5 parent, and the strains were evaluated by performing secretion assays as described in Figure 3. Proteins were visualized by immunoblotting with antibodies to β-lactamase (YopM-Bla), YopM, YopE (TTSS secreted proteins), YscD, YscF (TTSS apparatus components) Ail (outer membrane protein) and RpoA (cytoplasmic protein, loading and fractionation control). An asterisk indicates truncated Yops due to degradation by Pla protease.

Since several mutants displayed defective expression of TTSS proteins, and since the defect in secretion could be bypassed by cell contact in some cases to allow Yop injection during cell culture infection, we hypothesized that there may be conditional blocks in the TTSS at the level of expression. To determine if heterologous expression of a Yop could bypass the defects observed in the mutants, we introduced a plasmid carrying a *yopM-bla* fusion located behind the constitutive *nptII* promoter. As previously demonstrated for other Yop fusions under the control of the this promoter [51]#, expression of YopM-Bla was considerably lower than from the native *yopM* promoter (Figure 5). Despite the low level of expression, YopM-Bla and its characteristic degradation products were routinely detected in the supernatant of wild type secretion assays (Figure 5). For the CHI strains, all of them consistently expressed large amounts of YopM-Bla; however, the reporter did not appear in culture supernatants (Figure 5). It remains a formal possibility that low levels of Yops or YopM-Bla are secreted in these mutants, which are undetected due to Pla activity; however, after repeated attempts to observe secretion at various times after induction of the TTSS, we never observed YopM-Bla or any apparent degradation products of the reporter in the culture supernatants. This lack of secretion, despite high expression levels of the reporter, supports the conclusion that Yop secretion is impaired in these mutants. Therefore, it is unlikely that the absence of Yops in the supernatants during secretion assays (Figure 3 and 5) is simply due to detection limits of the assay or a result of impaired Yop expression. The defects may however be attributed to decreased expression of the TTSS machine components, at least in the cases of *folD^−^*, *pgsA^−^*, and *pssA^−^* (Figure 5), as these mutants display less YscD and YscF.

It is important to point out that while some Yops could be detected in the supernatant of *pgsA^−^* (Figure 5), the cytoplasmic protein RpoA, which is used as a fractionation control, was also found in the supernatant. In contrast, the majority of Ail (an outer membrane protein and additional fractionation control) was found in the cell pellet fraction, as expected. This was a consistent observation, even after filtering supernatants to ensure removal of cells, suggesting that these mutant cells may be leaky (Figure 3 and data not shown). Therefore, we cannot determine from these experiments whether the small amount of Yops occasionally seen in the supernatant for this mutant is due to type III secretion or simple leakage.

**Figure 6:**
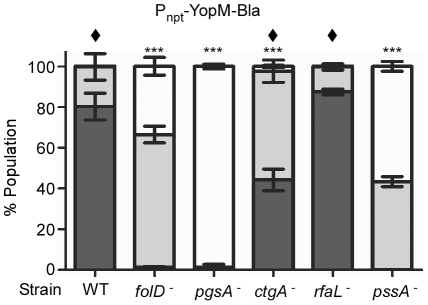
Translocation phenotypes of strains carrying a heterologously expressed Yop reporter. CHO cells were infected with *Y. pestis* strains carrying pAH83 at 37°C for 3 hours, followed by CCF2-AM staining and flow cytometry. Cells were analyzed for blue and green fluorescence. The level of translocated Bla reporter is indicated by white (none), gray (intermediate level), or black (high level) bars. For each experiment, the assays were performed in triplicate, and error bars indicate the standard deviation. The data shown is representative of three independent experiments. One-way ANOVA with Tukey post-hoc test was done to demonstrate significant differences in injection (high-level injection, black bars) for mutants relative to the wild type parent. *** *P*<0.001. Diamonds indicate statistically significant differences (*P*<0.001) compared to samples in Figure 4, Panel A.

Since cell contact was able to bypass the *in vitro* secretion defect for some mutants, we tested whether heterologous expression of the YopM-Bla reporter could restore injection levels to that of wild type for the transposon mutants. As expected, constitutive expression of YopM-Bla resulted in high levels of its injection into CHO cells by the parent strain (Figure 6). Again, *rfaL^−^* displayed a similar ability to inject the YopM-Bla reporter. In fact, there was no significant difference in Bla reporter injection between wild type and *rfaL^−^* during any of the translocation assays (Figures 4 and 6). Both strains showed a statistically significant increase (diamonds: *P*<0.001) in YopM-Bla injection when the reporter was constitutively expressed (compare Figure 4A to Figure 6). Likewise, although injection of YopM-Bla by *ctgA^−^* was still impaired (*** *P*<0.001) compared to wild type (Figure 6), constitutively expressing the reporter did significantly enhance its injection (Figure 4A and 6). In contrast, *folD^−^*, *pgsA^−^*, and *pssA^−^* did not respond to heterologous expression of the reporter. Both *folD^−^* and *pssA^−^* translocated similar amounts of the YopM-Bla reporter whether expression was driven from the native promoter or the *npt* promoter, and *pgsA^−^* did not translocate any YopM-Bla.

### Role of folate synthesis on type III secretion

**Figure 7:**
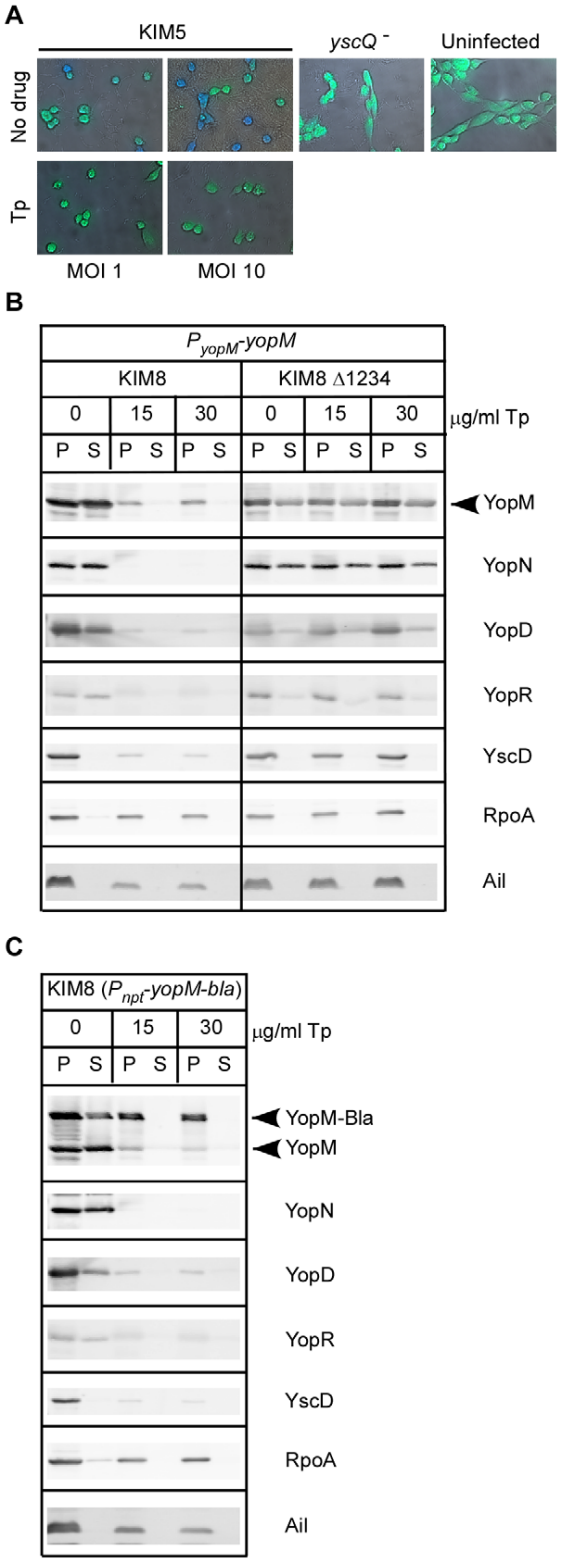
Trimethoprim inhibits the *Yersinia* TTSS. **A.** HeLa cells were infected with KIM5 or CHI 30 (*yscQ* ^−^) expressing YopE-Bla. Infections were carried out at the indicated MOI for three hours in the presence or absence of 500 µg/ml of trimethoprim. Following infection, cells were stained with CCF2-AM and visualized by microscopy to assess translocation of the YopE-Bla reporter. Blue fluorescence = YopE-Bla injection; green fluorescence = no injection. **B.** *Y. pestis* KIM8 and KIM8 Δ1234, carrying pMM207 (*yopM* expressed from native promoter), were subcultured into MM9 and grown at 26°C for 2 hours. Trimethoprim was then added at the indicated concentrations (0, 15, or 30 µg/ml) and the cultures were shifted to 37°C for 3 hours, followed by TCA precipitation of supernatant (S) and cell pellet (P) fractions and immunoblotting with antibodies against YopM, YopN, YopD, YopR (TTSS secreted proteins), YscD (TTSS machine), RpoA (cytoplasmic protein), Ail (outer membrane protein). Degradation products are not indicated since KIM8 and KIM8 Δ1234 lack Pla protease. **C.** Secretion assays and western blotting was performed as in Panel B using *Y. pestis* KIM8 carrying pAH83 (*yopM* expressed from *nptII* promoter).

One of the mutants, CHI 919 (*folD^−^*), was unable to secrete Yops *in vitro* and was only able to inject low levels of Yops into tissue culture cells (Figures 3,4,5,6). To determine whether the effect of the Mu insertion in CHI 919 on type III secretion was specific to the *folD* gene, or if it was reflective of folate biosysnthesis in general, we tested whether trimethoprim, a dihydrofolate reductase (DHFR) inhibitor, also inhibits injection of Yops by the TTSS (Figure 7). When the wild type strain KIM5 was incubated for three hours with trimethoprim, bacterial growth was inhibited, but the cells were still viable throughout the duration of the experiment (data not shown). We then infected HeLa cells with KIM5 carrying pMM85 (YopE-Bla), in the presence or absence of trimethoprim, for three hours. Because of the growth inhibition that was observed in the presence of drug, we used an MOI of 1 and 10 for the KIM5 infections. As a negative control, HeLa cells were also infected at an MOI of 10 with the TTSS mutant (*yscQ* ^−^) expressing YopE-Bla. After the infections, cells were stained with CCF2-AM and visualized by microscopy to evaluate injection of the Bla reporter. In the absence of drug at an MOI of 10, large numbers of bacteria could be seen, and most of the HeLa cells displayed blue fluorescence indicating translocation of the YopE-Bla reporter (Figure 7A). When the MOI was decreased to 1, in the absence of drug, there was a mixture of green and light blue cells indicating that the YopE-Bla reporter was injected into host cells to a lesser extent. In contrast, when trimethoprim was added, no blue cells were observed, even at an MOI of 10, which gave a similar bacterial density to infection at an MOI of 1 in the absence of drug (Figure 7A). Likewise, cells that were either uninfected or infected with the *yscQ* ^−^ mutant did not turn blue, confirming that the appearance of blue cells correlates with delivery of YopE-Bla in a TTSS dependent manner. Because no injection of YopE-Bla was observed for cells treated with trimethoprim, we hypothesized that trimethoprim may prevent expression and/or function of the TTSS.

To further assess the effects of trimethoprim on the TTSS, we conducted secretion assays in MM9 in the presence or absence of trimethoprim (0, 15, 30 µg/ml). For these experiments, KIM8 (*pla* ^−^) was used as the parent strain to ensure that the absence of secreted Yops is not due to degradation by the Pla protease. We also used *Y. pestis* strain KIM8 Δ1234 [37], which carries a dihydrofolate reductase cassette conferring trimethoprim resistance, thereby allowing us to assess any non-specific effects of the drug on protein expression.

This strain lacks all the effector Yops (Δ*yopM/yopT/sycT/yopK/ylpA/yopE/sycE/ypkA/yopJ/yopH*), so we introduced a plasmid carrying the *yopM* locus into both KIM8 and KIM8 Δ1234. This allowed us to track expression and secretion of YopM, as well as YopN, YopR, and YopD, which are still present in KIM8 Δ1234. We also tracked expression of two non-TTSS proteins: Ail, which is an outer membrane protein adhesin that is constitutively expressed [52], [53], and RpoA As shown in Figure 7B, expression of TTSS proteins (YopM, YopN, YopR, YopD) was severely reduced after addition of the drug to the KIM8 parent. Not surprisingly, secretion of those proteins was also severely reduced (at 15 mg/ml) or abolished (at 30 mg/ml) of trimethoprim. However, expression of unrelated, non-TTSS proteins (Ail and RpoA) was only slightly reduced in the presence of the drug. In contrast to KIM8, expression and secretion of all proteins by KIM8 Δ1234 was unaffected even at the highest concentration of trimethoprim (Figure 7B).

Since expression of non-TTSS proteins was less affected by trimethoprim treatment, we asked whether heterologous expression of a Yop could overcome the affects of trimethoprim. We therefore introduced pAH83, which expresses YopM-Bla from the *nptII* promoter, into KIM8 and performed secretion assays as before. As shown in Figure 7C, expression of YopM-Bla from the *nptII* promoter was also only slightly reduced, even at the highest trimethoprim concentration. However, despite being expressed, YopM-Bla was not secreted in the presence of trimethoprim, which is probably due to the lack of TTSS proteins. These observations suggest that availability of folate contributes to the regulation of type III secretion in *Y. pestis* and may be reflective of a link between the TTSS and the metabolic state of yersiniae. Furthermore, since type III proteins were either absent or barely visible within the pellet fractions, it suggests that the effect of a block in the DHFR pathway is at the level of expression of genes in the type III pathway.

### Independent mechanisms for activating the TTSS

**Figure 8:**
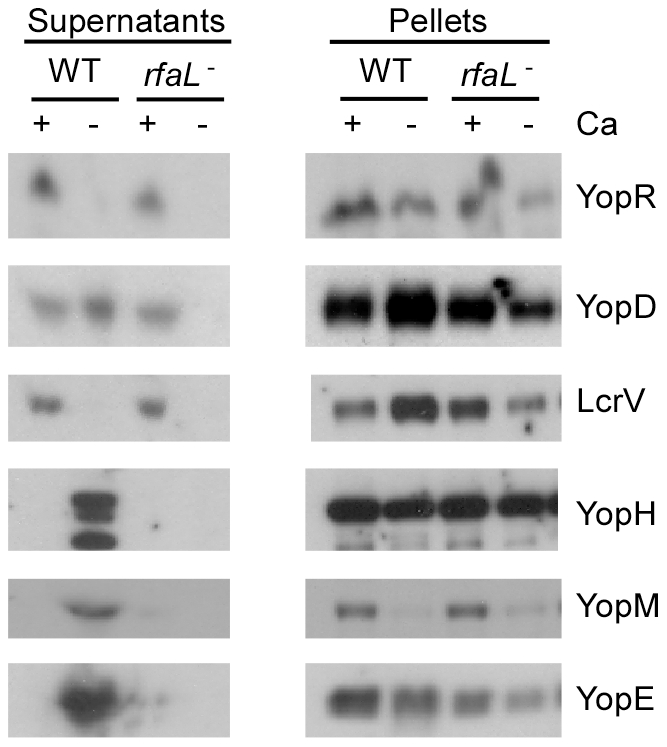
CHI 1800 cannot induce type III secretion in response to low calcium. *Y. pestis* strains KIM5 and CHI 1800 (*rfaL* ^−^) were subcultured into DMEM with or without EGTA and grown at 26°C for 2 hours and 37°C for 3 hours, followed by TCA precipitation of supernatant and cell pellet fractions and immunoblotting for early Yops (YopR, YopD, LcrV) and late Yops (YopH, YopM, YopE).

One of the transposon mutants, CHI 1800 (*rfaL^−^*), despite having a secretion defect *in vitro* (Figures 3 and 5), was able to inject Yops like wild type during infection of CHO cells (Figures 4 and 6). The ability of *rfaL^−^* to inject Yops as well as the parent was independently verified using HeLa cells, followed by digitonin fractionation of infected cells and immunoblotting (data not shown). The finding that the *rfaL^−^* mutant is able to deliver Yops into host cells is surprising given its inability to secrete Yops in the absence of calcium *in vitro*. To determine whether the disparity in observed phenotypes could be attributed to the different growth media used in the assays, we conducted *in vitro* secretion assays in the same medium used to culture HeLa cells (DMEM supplemented with FBS and glutamax). As observed in Figure 8, the mutant was able to both express and secrete the early Yops YopR, YopD, and LcrV as well as wild type when grown in DMEM containing calcium. As expected the late Yops YopE, YopH, and YopM were not secreted in the presence of calcium by either wild type or mutant. However, when EGTA was added to chelate the calcium, YopE, YopH, and YopM were all secreted by wild type KIM5 (Figure 8). In contrast, when calcium was chelated from the medium, *rfaL* ^−^ did not secrete either early or late Yops. The mutant expressed all Yops in the absence of calcium, but several of the Yops appear to be synthesized at lower levels in the mutant compared to wild type (see pellet fractions of YopR, YopD, LcrV, YopE). In addition, the level of the TTSS component YscD is also reduced in the mutant in the absence of calcium (Figure S1). As expected the defects in expression and secretion are restored by complementing the mutant *in trans* (Figure S1). Since the expression levels of Yops and the TTSS machine are reduced when calcium is depleted, it is possible that the absence of calcium prevents the proper function of assembled needles in *rfaL* ^−^, leading to the negative feedback on TTSS expression that is typically seen for mutations within TTSS components. The data suggest that although the secretion apparatus in the *rfaL* mutant is capable of transporting early Yops, it cannot respond normally to the low calcium signal in order to fully activate the TTSS to secrete the effector (late) Yops, and this signaling defect can be overcome during cell contact. Thus, it appears that, while the type III pathway can be induced by either chelating calcium *in vitro* or by cell contact *in vivo*, *Y. pestis* can discern and respond to these two stimuli by different mechanisms.

### Virulence defects of CHI strains

The *Yersinia* TTSS is an essential virulence component, and mutants lacking a functional TTSS are unable to cause acute disease in mouse models of infection. Therefore, we next investigated the ability of the chromosomal mutants to cause disease in mice. BALB/c mice were infected intravenously with the KIM5 parent or its isogenic mutant CHI strains. Doses were administered in 10-fold increments spanning from 10^0^ to 10^5^ cfu for wild type and from 10^2^ to 10^7^ for the mutants in order to determine the AD_50_ (dose required to cause acute disease in 50% of the population). The results are summarized in Table 1. As expected, wild type KIM5 produced signs of disease in a dose dependent manner. For mice infected with the highest dose (10^5^ cfu) visible symptoms, such as ruffled fur and squinty eyes, were apparent on day 1. The disease progressed normally and by days 5–6 signs of terminal disease were apparent (severely matted fur and partially or completely closed eyes, severe weight loss, rigid hunched posture, lack of mobility even when touched, labored breathing), at which point the mice were judged to be moribund and were euthanized. The AD_50_ for the parent was determined to be between 800–3400 cfu in three independent experiments. In contrast, all mutants were reduced in virulence by several orders of magnitude. Infections at the highest dose of 10^7^ cfu with *folD^−^*, *pgsA^−^*, and *pssA^−^* all resulted in the appearance of ruffled fur for 2 to 3 days post-infection, indicating illness relating to the high doses of Gram-negative bacteria. However by days 5 to 6, the symptoms of disease had disappeared and all mice survived. No mice were ever moribund and no deaths occurred at these doses or any of the lower doses for infections with any of these mutants. Identical results were obtained using the *yscQ* TTSS mutant isolated from this screen. These results demonstrate that these three mutants are indeed avirulent.

**Table 1:** Mutant phenotypes.

Strain	Mutation	Doubling time (hours)^a^	AD_50_	Median survival^b^
KIM5	WT	1.34 (0.03)	800–3400 cfu	5.5 days
CHI 919	*folD*	1.95 (0.01)	>10^7^ cfu	ND
CHI 1065	*pgsA*	1.60 (0.03)	>10^7^ cfu	ND
CHI 1345	*y0447 (ctgA)*	1.31 (0.02)	4×10^6^ cfu	7 days
CHI 1800	*y3762 (rfaL)*	1.28 (0.09)	= 10^7^ cfu	7 days
CHI 1965	*pssA*	1.62 (0.03)	>10^7^ cfu	ND

a In vitro growth curves were done using Heart Infusion Broth.

b Median survival was calculated based on infections with WT at 10^5^ cfu, the *ctgA* mutant at 10^7^ cfu, or the *rfaL* mutant at 10^7^ cfu. No deaths occurred during infections with other mutants, so survival times are not indicated (ND).

**Figure 9:**
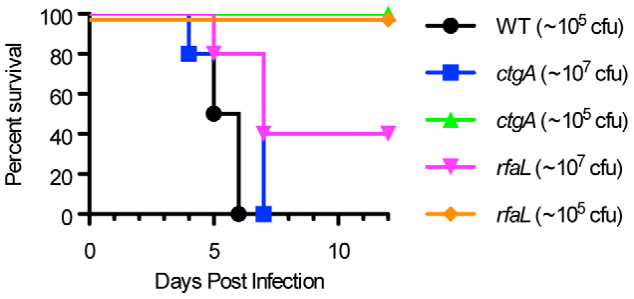
In vivo attenuation of CHI strains. BALB/c mice were infected intravenously with serial dilutions *Y. pestis* KIM5 or transposon mutants. Moribund mice were euthanized and time to death was recorded. Survival curves are shown for KIM5 (WT) and two mutants (*ctgA* ^−^ and *rfaL* ^−^).

In contrast, *ctgA^−^* and *rfaL^−^* were extremely attenuated but did retain some ability to cause disease at the highest doses tested. Five out of five mice infected with 10^7^ cfu of *ctgA^−^* became moribund and were euthanized by days 4 to 7. All mice infected with lower doses of *ctgA^−^* survived. From this, we estimate an AD_50_ of ∼4×10^6^ cfu. Similarly, all mice infected with 10^7^ cfu of *rfaL^−^* became ill, but only 3 of 5 mice progressed to moribundity and were euthanized 5–7 days post-infection. Mice infected with lower doses of *rfaL^−^* did not become ill. From this, we estimate that the AD_50_ is equal to or greater than 10^7^ cfu. Because it is not possible to test higher doses, we can only estimate an AD_50_ for these two mutants and therefore cannot determine whether there is statistically significant difference in virulence between them. However when the data is evaluated to determine time to death, there does not appear to be any significant difference. Figure 9 shows the survival curves for mice infected with 10^5^ cfu of WT, 10^5^ or 10^7^ cfu of *ctgA^−^*, and 10^5^ or 10^7^ cfu of *rfaL^−^*. The median survival time was 5.5 days for mice infected with WT, compared to 7 days for mice infected with either mutant at the 10^7^ cfu dose. The Log-rank test was used to compare the survival curves between WT and the two mutants. There was no significant difference between the two mutants, indicating that they are similarly attenuated (P = 0.2725). When each mutant (10^7^ cfu) was compared to WT (10^5^ cfu), there was also no significant difference (P = 0.0629 for *ctgA^−^* and P = 0.0361 for *rfaL^−^*), which indicates that at the highest doses tested the mutants resemble WT in their ability to cause terminal disease. Thus, while *ctgA^−^* and *rfaL^−^* display severely attenuated phenotypes in this mouse model of infection, these strains are not completely avirulent and retain some ability to cause lethal disease in mice.

## Discussion

The *Yersinia* TTSS is an essential component of virulence, and loss of the TTSS, either by mutational inactivation or loss of the pCD1 virulence plasmid, corresponds with a reduction in virulence by six orders of magnitude or more [54]#. Therefore, the TTSS represents an important target for vaccine and therapeutic approaches. Previous work alluded to the presence of chromosomal loci, which contribute to the regulation of type III secretion of Yops by *Yersinia pestis* [24]. However these genes have remained unidentified. We sought to identify these chromosomal loci as they represent potential targets for vaccine development.

### CHI 1065 *(pgsA) and CHI 1965 (pssA)*

After screening over 2000 strains carrying transposon insertions and displaying the LCR^−^ growth phenotype, we identified five mutants that displayed a strong defect in type III secretion. Three of those mutants (CHI 1065, 1800, 1965) carried insertions in genes involved in the biosynthesis or integrity of the cell envelope. CHI 1065 carries an insertion in *pgsA*, which encodes a phosphatidylglycerophosphate synthase. Phosphatidylglycerol (PG) is an essential component of membranes, and *E. coli* mutants lacking *pgsA* are not viable [55]. While it appears that *pgsA* is not an essential gene in *Y. pestis*, CHI 1065 does have a growth defect as shown by a ∼20% longer doubling time compared to wild type (Table 1). PG is essential for lipid modification and the proper localization and/or function of proteins such as DnaA and SecA. It is also a precursor for membrane-derived-oligosaccharides (MDO's), which serve as an osmoprotectant. In *Y. enterocolitica*, the lipoprotein VirG [56] is required for the proper localization of the YscC secretin in the outer membrane [57]. The lipid modification of VirG is essential for its interaction with YscC and is therefore necessary for type III secretion of Yops [58]. Because of the involvement of PG in localization of membrane proteins, and its requirement for the Sec-dependent translocation pathway, it is likely that the effect of the *pgsA* mutation in *Y. pestis* is pleiotropic. Further work is necessary to determine if components of the type III secretion machinery, as well as other membrane proteins which may indirectly contribute to Yop secretion, are mislocalized in CHI 1065.

Likewise, the *pssA* mutation in CHI 1965 may affect membrane structure or localization of membrane proteins. *pssA* is required for the synthesis of phosphatidylserine (PS) and subsequently phosphatidylethanolamine (PE), which comprises 70–80% of membrane phospholipids [55]. PE constituents also decorate MDO's and LPS, thereby potentially contributing to membrane structure and integrity. As in the case of *pgsA^−^*, the loss of *pssA* function is not a lethal event, but it does result in approximately 20% longer doubling time *in vitro* (Table 1).

### CHI 1800 (*y3762* or *rfaL*)

Surprisingly, in CHI 1800 we identified an insertion in a gene predicted to be involved in LPS biosynthesis (*y3762*). Although *Y. pestis* harbors several mutations in the O Antigen biosynthetic cluster [59], [60], it does produce a lipo-oligosaccharide (LOS) with pyrogenic properties [61]#. The core component of *Y. pestis* LOS is based on a pentasaccharide containing 4 heptose residues attached to lipid A via Kdo [62]. CHI 1800 carries an insertion in *y3762*. Although there were no BLAST hits with any significant homology, *y3762* is annotated as a putative lipid A-O Antigen ligase. Further comparison of Y3762 and RfaL (the corresponding protein in *E. coli* [63]#) reveals that both of these proteins are largely transmembrane and the largest contiguous periplasmic stretch of the proteins contain the O-antigen polymerase domain. Moreover, *y3762* is located in a predicted operon with *rfaQ*, which is involved in completion of the inner core of LPS in *E. coli*. Therefore, we propose to refer to *y3762* as *rfaL*.

The lack of O Antigen in *Y. pestis* makes the physiological function of RfaL unclear. Interestingly, in *E. coli* RfaL is also involved in the transfer of alternative saccharides, including enterobacterial common antigen (ECA), to the LPS core [64], [65]. The *Y. pestis* genome carries the genes for ECA biosynthesis, and several mutations in that pathway were isolated in our screen that lead to only a slight decrease in Yop secretion (unpublished observations). Therefore RfaL may be required for the attachment of an unidentified saccharide moiety to the LOS core.

### Low calcium response versus cell contact

Interestingly, *rfaL^−^* exhibited the ability to distinguish between activation of the type III pathway in response to low calcium versus cell contact. A similar phenotype was observed in *Pseudomonas aeruginosa*, where it was found that there are two independent pathways for inducing expression of TTSS genes [66]#. These observations suggest that although a low calcium environment can trigger secretion, it is not a mimic of cell contact. Additionally, our results indicate that there are different mechanisms for activation of the secretion machinery in *Y. pestis*. It is possible that chelating environmental calcium induces a conformational change in the secretion apparatus, thereby allowing secretion substrates to pass. In the context of a cell culture infection, where there is calcium present in the medium, protein-protein interactions during cell contact could also lead to a conformational change leading to secretion of TTSS effectors. Further work is required to determine how the conformational change in the secretion apparatus is sensed and which proteins are involved in the hypothetical protein-protein interaction during target cell contact.

### CHI 1345 (*y0447* or *ctgA*)

One of the mutants, CHI 1345, isolated in this screen carried a mutation in a hypothetical gene. Y0447 is predicted to have 4 transmembrane helical domains and localize to the cytoplasmic face of the membrane. It is annotated as belonging to the DedA family of membrane proteins. Proteins from this family can be found in members of the α-, β-, and γ- proteobacteria. Little is known about the function of the proteins, and these proteins often share little similarity in their amino acid sequences. However, screens performed in *E. coli*, *Salmonella*, and *Neisseria* have shown that proteins in the DedA family are associated with cell division, response to divalent cations, and resistance to cationic antimicrobial peptides, all of which are linked to membrane biology [67], [68], [69]. The phenotypes of the mutants isolated in these screens suggest that they may have structural defects in their membranes. Mutations in genes encoding YghB and YqjA of the DedA family in *E. coli* result in an altered phospholipid ratio in the membranes of the mutants [67]. Interestingly, CHI 1965 (*pssA* ^−^) and CHI 1065 (*pgsA* ^−^) isolated from our screen would also be predicted to have altered phospholipid ratios. We suspect that *y0447*, *pgsA* and *pssA* may play a role in maintaining the structure and stability of the bacterial membranes. Defects in membrane biology in these mutants could consequently affect the structure or stability of the TTSS machinery. It is not clear at this point if any such membrane defect would prevent the proper assembly of the TTSS machinery or if these alterations would activate cellular stress response systems that could lead to a regulatory block on expression of the TTSS genes. Because this is the first characterization of *y0447*, we will refer to it as *ctgA (chromosomal type III secretion gene)*, for its role in type III secretion.

### CHI 919 (*folD*)

In contrast to several other screen isolates with mutations affecting membrane structure, the transposon insertion in *folD* in CHI 919 likely causes an effect on metabolic functions. *folD* is annotated as a bifunctional enzyme (5, 10-methylene-tetrahydrofolate dehydrogenase and cyclohydrolase) similar to that of *E. coli* [70]# which is involved in tetrahydrofolate (THF) production. The *Y. pestis* genome harbors two additional genes, *glyA* and *gcvT*, whose products are predicted to bypass the FolD-catalyzed reactions, thus explaining the viability of CHI 919 in media lacking folate. Despite this, CHI 919 had the most severe growth defect of the mutants with a ∼65% longer doubling time (Table 1). THF is an important coenzyme and provides one-carbon donors to several metabolic pathways including purine, methionine and thymidylate synthesis [71]. The observation that trimethoprim inhibits type III secretion (Figure 7), likely at the level of expression, confirms the importance of *folD* and/or THF in type III secretion. Therefore, the lack of secretion in a *folD* mutant (CHI 919) may reflect a connection between type III secretion and the metabolic state of the cell. This hypothesis is supported by studies in *Pseudomonas aeruginosa*, which showed that histidine levels and the two-component system, *cbrAB*, contribute to the regulation of type III secretion [72]. Similarly, the *aceAB* operon, which encode subunits of pyruvate dehyrogenase, are also necessary for type III secretion in *P. aeruginosa* [73].

### Perspectives

In conclusion, *Y. pestis* genes identified in a Mu insertional mutagenesis screen for LCR phenotypes point to hitherto unappreciated roles for LPS biosynthesis, envelope structure and integrity, and intermediary metabolism in the *Yersinia* TTSS. Further, unknown genes that have never been linked to type III secretion may reveal novel regulatory or assembly mechanisms for the type III pathway. Several other genetic screens using STM or IVET to identify virulence genes have been conducted in *Yersinia* species [74], [75], [76], [77], [78], yet there is no overlap between the genes identified previously and those identified in our study. One possible explanation is that we did not saturate the screen, and presumably there are additional chromosomal genes that may contribute to type III secretion. Additionally, the STM and IVET screens are used to identify genes that are important *in vivo*, and the approaches are fundamentally different than ours. It is interesting to note that all of the mutants tested in our screen demonstrated a clear virulence defect. Perhaps a more thorough STM screen in *Y. pestis* would also identify these or similar genes. In *P. aeruginosa*, several screens have identified environmental sensors and two-component systems, such as *soxR*, *copRS*, and *retS*, as important regulatory factors [79], [80], [81], [82]. Although we did not identify any of those genes in this study, it is not unlikely that similar signal transduction cascades exist in *Yersinia* species, and this is a prospect that warrants further investigation.

Finally, as one goal of our screen was to identify candidates for vaccines or new antimicrobials, we must point out the affect of trimethoprim on the TTSS (Figure 7). Trimethoprim, which is among the treatment options for plague infections, targets FolD. And while the antimicrobial properties of trimethoprim are independent of its affect on the *Yersinia* TTSS, the isolation of a *folD* mutant (CHI 919) in our screen along with the observation that trimethoprim prevents proper expression of TTSS components, may indicate an additional indirect method for the drug to inhibit disease progression. It is possible that other genes identified by this study would also make good targets for antimicrobials. A particularly interesting possibility for a broad-spectrum target is CtgA, which appears to be widely conserved. Since loss of CtgA impairs TTSS without affecting growth, targeting it may be useful in reducing virulence without putting a direct selective pressure on growth and correspondingly on the selection of drug-resistant mutants.

## Supporting Information

Figure S1 **Secretion phenotype of CHI 1800.** *Y. pestis* strains KIM5 and CHI 1800 (*rfaL* ^−^), in the presence or absence of complementing plasmid, were subcultured into DMEM with or without EGTA and grown at 26°C for 2 hours and 37°C for 3 hours, followed by TCA precipitation of supernatant and cell pellet fractions and immunoblotting for YopE and YscD
(TIF)

Table S1 **Primers used in this study.**
(DOC)
