# Supplementary figures and images for: Identification of Chromosomal Genes in Yersinia pestis that Influence Type III Secretion and Delivery of Yops into Target Cells
Source: PLoS One. 2012 Mar 30;7(3):e34039. doi: 10.1371/journal.pone.0034039 (PMC3316589; doi:10.1371/journal.pone.0034039)

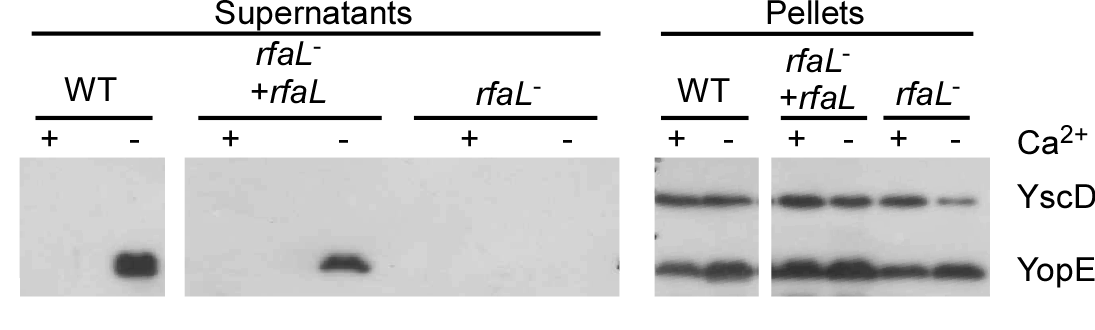

Supplement: Figure S1 — Secretion phenotype of CHI 1800. Y. pestis strains KIM5 and CHI 1800 (rfaL −), in the presence or absence of complementing plasmid, were subcultured into DMEM with or without EGTA and grown at 26°C for 2 hours and 37°C for 3 hours, followed by TCA precipitation of supernatant and cell pellet fractions and immunoblotting for YopE and YscD (TIF) [file pone.0034039.s001.tif]
