# Supplementary material for: Identification of Chromosomal Genes in Yersinia pestis that Influence Type III Secretion and Delivery of Yops into Target Cells
Source: PLoS One. 2012 Mar 30;7(3):e34039. doi: 10.1371/journal.pone.0034039 (PMC3316589; doi:10.1371/journal.pone.0034039)
Supplement: Table S1 — Primers used in this study. (DOC) [file pone.0034039.s002.doc]

TABLE S1. Primers used in this study.

|  | Primer name | Primer sequence |
| --- | --- | --- |
| iPCR and sequencing | BfaI-LT | tgcactacaggcttgcaagccccac |
|  | BfaI-RT | agctggttcatggttgttatacggc |
|  | TaqI-LT | ccgtaaatacacgcctcgttgtcca |
|  | TaqI-RT | ccataccctaagtgatccccatgta |
|  | Taq-RT-nested | gctaaagttttcgcatttatcg |
|  | Bfa-LT-nested | cagatcccgaataatccaatg |
| Gene complementation |  |  |
| *folD* | npt.pro.SOE.PstI.1 | ctgcaggcgcaagggctgctaaag |
|  | npt.pro.folD.SOE.2 | cgcaatcgttttaccatcaataatttttgctgacatgcgaaacgatcctcatcctgtctc |
|  | folD.SOE.3 | gagacaggatgaggatcgtttcgcatgtcagcaaaaattattgatggtaaaacgattgcg |
|  | folD.SOE.BamHI.4 | ggatcctcaattttggctgatgtcgtgatattcc |
| *pgsA* | pgsAORFNdeI | catatgcaattgaatataccgac |
|  | pgsAORFBamHI | ggatcctcaaggttcgagcaaatcac |
|  | uvrYproEcoRI | gaattccgtgacattcatcatctagg |
|  | uvrYproNdeI | catatgagaaatttctccagaaataagggag |
| *ctgA* (*y0447*) | y0447attB1 | attB1-cggctaaaggctacaacctg |
|  | y0447attB2 | attB2-ctagccaccaataacgtcaa |
| *pssA* | npt.pro.pssA.SOE.2 | gtttgctacgtttgaattttgacaacatcacgcgaaacgatcctcatcctgtctc |
|  | pssA.SOE.3 | gagacaggatgaggatcgtttcgcgtgatgttgtcaaaattcaaacgtagcaaac |
|  | pssA.SOE.revEcoRI | gaattcttatagaatacggctaattaaccgatcaatgcgg |
| *rfaL* (*y3762*) | y3762attB1 | attB1-ctctttggaaggctccttgc |
|  | y3762attB2 | attB2-tctgaatgtgtattgcgtct |
|  | attB1 | aaaaagcaggcttc |
|  | attB2 | agaaagctgggtc |
|  | attB1 adapter | ggggacaagtttgtacaaaaaagcaggct |
|  | attB2 adapter | ggggaccactttgtacaagaaagctgggt |
| YopM Reporters |  |  |
| pAH83 | npt.pro.yopM.SOE.2 | tttcttggatttatgaacatgcgaaacgatcctcatcctgtctc |
|  | npt.pro.yopM.SOE.3 | gagacaggatgaggatcgtttcgcatgttcataaatccaagaaatgtatctaat |
|  | yopM.SOE.4.bla.2 | ttcaccagcgtttctgggtgctcaaatacatcatcttc |
|  | bla.SOE.4.EcoRI | gaattcttaccaatgcttaatcagtgagg |
|  |  |  |
| pMM207 | YopM upstream | ggatccggtcagactcgatacgatta |
|  | YopM downstream | aagcttggtatgatagaatataaattctatg |
|  |  |  |
